# Cases of Peripheral Paralysis, Their Causes and Nature

**Published:** 1874-02

**Authors:** S. G. Webber


*Cases of Peripheral Paralysis, their Causes and Nature.* By S. G. WEBBER, M.D.

The nerves are generally protected from slight injuries by their situation. This is not, however, universal, and several nerves pass near the surface or over the bony prominences, so as to be more or less exposed to injury. Of course, severe injuries may affect nerves as well as other structures. The protection afforded by being on the inside of a joint, as in the axilla, fails in case of dislocation, and the head of the displaced bone may seriously injure the neighboring nerves.

Symptoms arising from pressure vary greatly, according to the severity with which the cause acts and the length of time during which the pressure is exerted. This probably explains a difference of opinion in regard to the causation of paralysis of the radial nerve. Duchenne, in his second edition, recognizes only a rheumatic origin. Panas, in a memoir read in 1871 before the Academie de Medecine and lately published,* refers all such cases to pressure as the cause. Duchenne, in the third edition of his work, refers to Panas' memoir, and says: "I admit that, in some cases, compression of the posterior part of the fore-arm or of the radial nerve should exercise a certain influence; but it seems to me un-

* Archives Generales de Medecine. Juin, 1873.

questionable that, in the numerous clinical cases which I have observed for more than twenty years (about one hundred), the action of cold has played the chief part in the etiology of this form of paralysis."

The radial nerve is not the only one which suffers from pressure or the action of cold, and it is not unlikely that all such cases agree in symptoms more closely than is apparent from published records, the intensity of some symptoms varying in different cases.

When nerves are divided, the peripheral end and the muscles which they supply degenerate; after reunion of the nerve, restoration, more or less complete, takes place. The electrical reaction of the nerves and muscles suffers a corresponding change, which is characteristic. A few days after the injury, the muscles lose their sensitiveness to the action of the faradic or induced current, and this loss increases until the strongest faradic current, applied by means of moist electrodes to the skin over the muscle or its nerve, no longer excites contraction. When a nerve is entirely separated from its centre, this extreme limit of change is reached within two weeks. In many cases, not all, the reaction of the muscle to the galvanic current is increased, while that for the faradic is diminishing. Later, as the degenerative change in nerve and muscle advances, the reaction to the galvanic current diminishes and finally disappears. With this, there is more or less atrophy of muscles.

When the nerve is restored, the reaction to the galvanic current usually reappears first, then voluntary motion, and, finally, the reaction to the faradic current. This order of return to normal conditions is not invariable.

If only a few fibres of a nerve trunk are injured, only a partial paralysis will follow, unless subsequent inflammation involves the whole trunk. One muscle alone may be paralyzed, or, if there is more than one source of innervation, it may be only weakened, and may still respond to the will and to electricity, though less perfectly than in health. If, instead of division of fibres, there is only a slight injury, every grade of change in their functional relations may be found from normal to entire loss. To this, and perhaps owing to hasty observation, is probably due the difference in opinion to which reference has already been made.

The means by which nerves may be compressed are various: a crutch pressing on the nerves in the axilla is not uncommonly the agent by which the compression is made; a wide board carried under the arm may act in the same way; one writer mentions badly fitting corsets; a dislocated bone, humerus, or femur, or other bone, may press on the nerves in the neighborhood of the joint; certain positions taken by the limbs during sleep, or the pressure of one part on another, as the head on the arm; a bandage applied tightly to a limb,—these are ways in which pressure, sometimes very gentle, but long-continued, may cause paralysis. A fall or a blow may exercise a more violent pressure of short duration, and is not unfrequently the cause of local paralysis and wasting. As the nerve is more severely bruised, and in part or entirely ruptured, compression is rather too mild a term, and contusion describes the injury better; but sometimes it is not easy to decide whether compression or contusion is the better. Often, in such cases, the muscles are paralyzed and suffer atrophy as a direct result of the injury.

That the severity of the cause producing the lesion has a controlling influence over the change in the electrical reaction, is the probable explanation of the difference in the following cases:

CASE I.—D. C., aged 23, fell, about seven weeks before I saw him, bruising his shoulder and breaking his collar bone. The deltoid and biceps were extensively wasted; there was inability to raise the arm or to flex the elbow. The muscles of the forearm were not affected. The triceps brachialis was scarcely at all implicated. The strongest faradic current the patient could bear caused very little if any contraction in the deltoid and biceps. The skin covering these muscles was very insensitive. The patient was seen only a few times, and gave up the treatment by electricity, saying he did not have time to attend. The atrophy of the deltoid may have been due to direct violence; that of the biceps, and the slight injury to the triceps, were probably secondary.

CASE II.—Lizzie B. was first seen early in April. About January 1st, she fell, striking her shoulder. The arm felt lame, but was not very painful. Immediately after, she was attacked with variola, and only three weeks later was it discovered by her physician that the arm was out of place. He replaced it, and the arm remained bandaged for five or six weeks. As nearly as could be learned from her statement, it would seem that immediately after the fall she had some power over the arm, but while sick and before the bandage was put on, this was lost and was not recovered.

She first applied at the City Hospital as an out-patient, and Dr. Bolles sent her to me.

When seen, the fingers were weak, the thumb and forefinger being weaker than the others. All the muscles of the forearm responded, however, to the will. The elbow could be bent and nearly straightened. The only motion of the arm at the shoulder-joint was a slight pendulum-like swing backwards and forwards. The shoulder could be raised a little, as in a shrug. There was a sense of pricking just over the head of the humerus and at the elbow, not in the fingers. There was lessened sensibility over the shoulder and on the outside of the arm. Sensation inside of the arm, over forearm and hand was normal. There was a "hard ache" in the shoulder, so severe as to keep her awake at night.

The deltoid, supra- and infra-spinatus, and perhaps the serratus magnus, were atrophied, especially the anterior part of the deltoid, causing the head of the humerus to project so prominently as to raise the question at first as to whether it was in its proper place. All the muscles responded fairly well to electricity, making allowance for their diminished bulk.

Galvanism was used, the positive pole being placed on the back of the neck, and the negative on the nerves, and stroked over the muscles. Passive motion was also used, and she was directed to rub the shoulder. The pain became less, so that she could sleep better. After three applications of electricity, she could use the arm to sew, could pin her cloak, and could raise the arm somewhat from the side.

She attended only very irregularly, being away for many days out of town. The last record was made after nine applications of electricity, more than a month after the first visit. The deltoid had then increased in bulk, covering the acromion and head of the humerus much more than at first. Motion was possible in all directions, but the arm, with forearm bent at right angles, could be rotated outwards only about one-half, and could be raised only to a horizontal. She could sweep, sew and iron, but had not strength enough to wash.

It may be a question as to whether the humerus was dislocated, or whether the atrophy of the deltoid caused it and the acromion to become so prominent as to simulate a dislocation; also, whether the restrained position of the arm had any part in the causation of the atrophy. This seems unlikely, as six weeks' disuse and the bandages necessary in case of dislocated humerus would not be likely to affect the deltoid. And she thought motion was lost before the bandage was applied.

While this case was under treatment, I saw another patient, a lady, who, from a fall three months previously, was suffering from inability to use her arm. The same muscles were affected, though the posterior fibres of the deltoid suffered more than the anterior. There were some symptoms of neuritis, and hyperæsthesia over the course of the nerves, with loss of faradic contractility, but preservation of susceptibility to the galvanic current. The treatment was short, owing to removal out of town, and but little advantage was gained from the two or three applications made.

In these cases, the cause was almost identical in nature, but undoubtedly varied in intensity. A similar variety in the symptoms, especially in the electrical reaction of the muscles, is found. In the first case, only seven weeks had elapsed from the time of the accident until I saw him, yet there was extensive atrophy and loss of electro-muscular contractility. In the second case, after three months, the latter was still retained; while in the third case, after three months, it was absent. In all, there was paralysis as a prominent symptom, with anæsthesia in two and hyperæsthesia in one.

Cases of paralysis from dislocation of the humerus are reported in medical writings. I have never seen a case, unless that of Lizzie B. is such. A while since, I was consulted by letter in regard to a patient suffering from such an injury. The deltoid and biceps were most affected, and there was partial anæsthesia over the deltoid.

Cases of paralysis following dislocation of the femur are rare. An interesting case is reported by Onimus and Legros.* A man, 46 years of age, received a mass of earth and stone on his left thigh. There was a dislocation, probably ilio-ischiatic, of the femur. There was severe pain, anæsthesia, and inability to move the leg. After reduction, the pain ceased, but the numbness remained, and he could not walk. After a slight attack of erysipelas of the face, pain in the leg returned. Five months after the accident, the left leg was found much emaciated, colder to the touch than the other, œdematous, especially around the tibio-tarsal joint. The leg could be feebly flexed and raised, the foot could not be raised nor the toes extended; toes flexed only partially. Sensation was much diminished; pressure was painful. Walking was not possible, and standing was painful. Electro-muscular contractility was very much enfeebled in all the flexor muscles of the leg, somewhat so in the extensors. It was diminished in the muscles of the calf, and gradually disappeared in the peronei and the tibialis anticus and extensor communis. Under the application of the galvanic current, the pain ceased, the œdema disappeared, and the patient could walk without a cane.

A much more common seat of paralysis is the radial nerve. It occurs, generally, during sleep. It is in regard to this paralysis that the difference of opinion between Duchenne and Panas, above referred to, exists.

Duchenne, in his third edition, says that the paralyzed muscles retain intact their electro-muscular contractility, and generally the muscular sensibility is not diminished. Once only has he observed enfeeblement of the cutaneous sensibility in the forearm and hand.

Panas also mentions the same characteristics, adding, however, that cutaneous sensibility may be perverted.

In the cases which have happened to come under my care, the electro-muscular contractility, as compared with the other side, has been found diminished when such comparison has been made at a long enough interval after the origin of the paralysis. If the two sides are not compared, the contractility remaining is sometimes so great that it might be considered normal; yet on making the comparison it is found more or less diminished. I have found the cutaneous sensibility for touch and temperature and pain almost

* Traite d'Electricite Medicale. Paris, 1872.

always diminished, or perverted, and for electricity sometimes increased. In the cases where paralysis has been most marked and existed longest, I have been able to see no difference in its nature or quality from that found in the cases due to severe lesion above recorded, excepting that the muscular atrophy was not marked enough to be recognized. Had the paralysis continued longer, it is possible that atrophy might have been found also.

In the number of this Journal for July 6th, 1871, I published four cases of this form of paralysis. In three, the electro-muscular contractility was diminished as compared with the other side; not mentioned in the other. Sensibility was diminished in three, perverted in the other. There was also some œdema of the hand, discolored surface, a mottled appearance and diminished heat as measured by the touch, once by the thermometer.

In several cases which I have since had under treatment, the same general characteristics have been observed in regard to sensation, coloration of skin, and apparent temperature. In only one has the electro-muscular contractility, as compared with the other side, been noted; the statement has merely been made that the muscles affected responded to faradization. In one case, it is noted that the muscles moving the thumb and index finger responded poorly to the faradic current, better to the galvanic.

Of the cases first published, in Case I, the paralysis came on while lying with the arm over the edge of a sofa in a drunken stupor; in Case IV, it came on during heavy sleep after great fatigue and a glass of whisky; in Case III, it came on as the direct result of carrying a basket of lemons on the shoulder and arm, the basket pressing on the outside of the arm, over the course of the radial nerve. Case II, may have been caused by a draught of cold air, in accordance with Duchenne's theory.

In the half-dozen recent cases, the paralysis was first noticed after sleeping. In only two was there any chance that cold was the cause. In one of these, seen in March, the patient said that, being warm, she threw off a part of her covering, but was not aware that she was chilled; she thought she lay on her arm, and slept rather heavily towards morning. The other was very slightly affected, and is for that reason reported.

Case III. Mrs. L. Seen with Dr. Ingalls. Had rheumatism ten years ago, and chronic arthritis since; at times pain in arm, especially during dull days. The joints were rather swollen. Cardiac enlargement with murmurs. The day before I saw her in the evening, she fell asleep in her chair; could not tell on which hand she leaned; slept less than forty-five minutes. On awakening, the left arm felt numb and prickly, and she had lost in part the use of the left hand. The sense of prickling passed off, but the power to use the hand did not return. The back of the hand, the thumb and fingers, were less sensitive than normal, the change being most marked in the thumb. The extensors were weak; pronation and supination were possible; flexion was slightly interfered with, there being a sense of stiffness. The hand was somewhat swollen, slightly congested, but not cold. All the muscles responded to the faradic current.

The effect was so recent, and seemed so slight, that only bathing in hot water and friction were advised. Recovery was perfect.

In this case, perhaps, time enough had not elapsed for change in the electrical reaction to appear. Whether pressure was the cause, or cold, it might be difficult to decide; certainly the symptoms resembled those found in other cases where pressure was undoubtedly the cause.

Taking the accounts given by Duchenne and Panas, it will be seen that several of the cases which I have reported differ in regard to the electro-muscular reaction, and all differ in regard to the condition of the cutaneous sensibility.

When a patient is seen the day after the origin of the trouble, it may well be supposed that the electrical reaction of the muscles would be normal, but after a few weeks have passed, it would probably be found, on comparing the two sides, that there is, almost always at least, a slight difference. The sensitiveness of the muscles to electrical stimulants varies much in different individuals, depending perhaps upon the amount of adipose tissue, the softness and suppleness, or dryness of the surface, and perhaps, more than all, upon the person's occupation. I have found the greatest susceptibility in teachers of the piano-forte. Hence, if only the affected arm is examined, the electrical contractility may be considered normal, when really it is diminished.

The cases which have come under my observation, where cold could be considered as the cause of the paralysis, were not essentially different from those where it was caused by pressure, unless it be that the electro-muscular contractility was either not affected or not tested as compared with the other side. In a case reported by Rosenthal,* which was probably caused by chill, it was decreased. In a case reported by Onimus and Legros, of so-called rheumatic paralysis of the anterior tibial nerve, it was likewise slightly diminished. In another case reported by them, the electrical reaction of the deltoid was below the normal for the faradic current, increased for galvanic.

It is hardly necessary to multiply instances.

As in all these cases the symptoms are identical, making allowance for variations in the severity with which the injurious agent acted, and the difference of time between the origin of the trouble and the examination, it is reasonable to consider the con-

* Electrotherapie, 2nd Edition, p. 269.

dition of the nerves, induced by these different agents, the same. It is only another instance where the same pathological condition is produced by different agents.

There is first pain, a peculiar pricking sensation, with this is numbness, and loss of motor power. The pricking sensations pass away, but the numbness and loss of power persist. The limb generally becomes œdematous, has a mottled or purplish color, and is frequently cooler than its fellow. To this may be added, in cases which are not seen soon after their origin, a diminution of faradic contractility in the muscles, sometimes the galvanic current acting more readily than normal. In recent cases, and in cases of slight intensity, some symptoms may be wanting.

A slight neuritis would explain all the symptoms.

The pricking is caused by irritation of the sensitive fibres, the numbness and loss of motor power by impaired conductility due to the inflammation. The œdema and discoloration, and the sinking of temperature, are due to implication of the vaso-motor fibres in the inflammation. The cause being slight, or of short duration, these symptoms are not found in their highest degree of development, and some cases recover easily; others, where the cause acted with more severity, are more protracted, and a few only result in atrophy and permanent disability.

Jaccoud, describing the symptoms of neuritis, gives as the principal, swelling of the nerve, no fever, pain following the course of the nerve, even to its terminal branches, increased by pressure, tactile sensibility diminished with persistence of spontaneous pain—painful anæsthesia. In mixed nerves, these symptoms are accompanied with startings and contractions of the muscles, to which soon succeed motor paralysis with loss of reflex movements and of the electric contractility. If the paralysis persists, muscular atrophy, lesions of nutrition, erythema, vesicular eruptions, and, in chronic cases, deposit of pigment and arthropathy. These symptoms are similar to those found in the cases already reported.

It would seem, from the consideration of the cases reported, that Duchenne is wrong in regard to the exciting agent of paralysis of the radial, and that, as Panas contends, it is generally caused by pressure. I cannot help thinking that both are wrong in regard to some of the important symptoms which are present in nearly every case, especially if it is of more than a week's duration. It is hardly necessary to refer to other authors, as these two represent the two phases of opinion, and most others refer to Duchenne. Eulenburg has, however, stated the symptoms correctly, and says that Duchenne is not correct in denying the loss of electro-muscular contractility. He also recognizes pressure during sleep as a cause.

Another cause of neuritis, which is not generally mentioned as such in text books, is overwork.

The following is an interesting case, showing as it does, in a mild degree, all the symptoms usually found in such cases. The patient was sent to me by Dr. Ingalls.

Case IV. Miss E. D., aet. 22, about three weeks before I saw her, while taking care of a brother sick with typhoid fever, noticed a tingling in the fingers of the right hand; they and the hand were also numb. These sensations occurred in the day time; there was no special strain or other cause for them, except the constant labor of nursing. With the numbness. was motor weakness. There was a steady increase of the affection. The pain was worse at night, and for a week began in the shoulder externally, and ran down to the fingers.

Two points could be distinguished as such, three-eighths of an inch nearer on right than on left back of hand, and on palmar end of right index finger one-eighth of an inch nearer. Sense of prick was more acute on the right, but the sense of mere contact with a blunt steel was more acute on the left.

There was a tenderness over the nerve, at the bend of the elbow, and over the ulnar nerve. The action of biceps and of the extensors in forearm was not easy, there being a sense of stiffness. The faradic contractility of the extensors of forearm was diminished; the galvanic contractility was increased, the muscles reacting to ten cells on the right, and to only fourteen, or perhaps thirteen, on the left. The flexors were not tested by electricity.

The galvanic current from fifteen cells was used, the positive pole being placed on the back of the neck, the negative passed over the muscles and held quietly over the nerves. After two applications, the faradic contractility of the muscles had improved. On the fourth visit, the sensation on back of the two hands was equal, and the same as it was on the left at the first visit. Ten days after I first saw her, it is recorded that she had no pain in the arm; it was strong and all right.

Soon after, other cases quite similar were seen, one seeming to originate from overwork in nursing, another from overuse of the arm in sewing and playing the piano, with, perhaps, a strain as predisposing cause; two others were from overwork in playing piano. In two cases, the electro-muscular contractility seemed increased on the affected side.

In some of these cases the affection of the nerve perhaps hardly deserved the name inflammation, possibly congestion would be more correct, there being, indeed, only the first stage of inflammation where several of the functions were increased rather than diminished.

Of paramount importance to the patient is the fact, that all these cases, so far as I know, where treatment has been commenced early and persisted in, have recovered. Where treatment was delayed, the cure was longer in being reached, but generally could be expected. The longest duration was two years before treatment; treatment extended through three months, with almost complete recovery.

The treatment consists in the application of either the faradic or galvanic current to the muscles and nerves affected. The galvanic current would probably be found more generally successful in a shorter time, though in many cases the other accomplishes all that is desired.

A few cases, as in Case III, may recover without electricity, but in most cases that agent will at least hasten a cure, and in other cases a cure has not been obtained after a year or more, without the use of the electricity, but followed soon after that agent was employed. It is not, then, always safe to wait for the unaided efforts of nature.—*Boston Med. and Surg. Journal.*

